# Optical Coherence Tomography Angiography Characteristics of Polypoidal Lesions in Caucasians

**DOI:** 10.1155/2023/9597673

**Published:** 2023-06-13

**Authors:** Daniel Ahmed-Balestra, Alexandra Graf, Martin Stattin, Anna-Maria Haas, Stefan Kickinger, Michael Jacob, Claus Zehetner, Katharina Krepler, Siamak Ansari-Shahrezaei

**Affiliations:** ^1^Karl Landsteiner Institute for Retinal Research and Imaging, Juchgasse 25, Vienna 1030, Austria; ^2^Department of Ophthalmology, Clinic Landstraße, Vienna Healthcare Group, Juchgasse 25, Vienna 1030, Austria; ^3^Institute of Medical Statistics, Medical University of Vienna, Spitalgasse 23, Vienna 1090, Austria; ^4^Department of Ophthalmology, Medical University of Innsbruck, Anichstraße 35, Innsbruck 6020, Austria; ^5^Medical School, Sigmund Freud University Vienna, Campus Prater Freudplatz 3, Vienna 1020, Austria; ^6^Department of Ophthalmology, Medical University of Graz, Auenbruggerplatz 1, Graz 8036, Austria

## Abstract

**Purpose:**

The aim of the study is to analyze the swept source-optical coherence tomography angiography (SS-OCTA) characteristics of polypoidal lesions in Caucasian patients.

**Methods:**

In this retrospective observational case series, 43 polypoidal lesions in 32 eyes of 32 patients were diagnosed using indocyanine green angiography (ICGA) and compared to SS-OCTA at a tertiary medical retina center (Clinic Landstra*ß*e, Vienna Healthcare Group, Austria) between June 2017 and March 2020. Vascularity was identified by color-coded B-scan SS-OCTA while morphology was described as revealed by en face SS-OCTA after alignment with ICGA-confirmed findings.

**Results:**

In total, SS-OCTA detected all polypoidal lesions, as identified by ICGA. On B-scan SS-OCTA, circumscribed flow was detected in 33 (76.7%) polypoidal lesions and diffuse flow in 10 (23.3%) lesions. On en face SS-OCTA, polypoidal lesions appeared morphologically as 19 tangled vessel balls (44.2%), 6 tangled vessel balls next to dilated vessels (13.9%), 8 vascular dilatations (18.6%), and 8 ill-defined vascular networks (18.6%), leaving 2 lesions (4.6%) undetected. Circumscribed flow was significantly associated with tangled vessel balls (*p* = 0.005).

**Conclusion:**

This study highlights the importance of a multimodal imaging approach, including SS-OCTA, for the evaluation of polypoidal lesions. Our findings suggest a morphological heterogeneity of vascular patterns in Caucasian patients with polypoidal lesions, as pictured by SS-OCTA.

## 1. Introduction

Polypoidal lesions were first described as a distinct macular disorder characterized by nodular excrescences with interconnecting vascular channels more than 30 years ago [1]. Later on, two basic choroidal vascular changes were identified with the help of indocyanine green angiography (ICGA): a branching neovascular network (BNVN) in the inner choroid and vascular dilations at the border of the vascular network [2]. Since then, ICGA has been considered the gold standard for the diagnostic evaluation of polypoidal lesions independent of their origin. More recently, optical coherence tomography (OCT) has become valuable as a dye-independent diagnostic tool [3]. However, OCT is limited to structural findings as it is unable to illustrate the vascular flow. The era of OCT angiography (OCTA)—commencing with the spectral domain (SD) technology—enabled the visualization of different vascular patterns such as round-shaped or cluster-like structures attributed to polypoidal lesions in a noninvasive setting [4, 5]. The introduction of swept source OCT angiography (SS-OCTA) with a deeper penetration and lower sensitivity roll-off facilitated further insights into the morphology [6–8]. Recently described in an Asian population, B-scan SS-OCTA demonstrated a high diagnostic accuracy of specific flow signals in polypoidal lesions inside or adjacent to pigment epithelial detachments (PED) [9]. In another study, polypoidal lesions appeared as tangled vascular structures in en face SS-OCTA [10]. In Caucasians, polypoidal lesions are most commonly associated with age-related macular degeneration (AMD) and the pachychoroid disease spectrum besides having an idiopathic appearance [11]. The observed features were reported to be similar to those in Asians using multimodal imaging without OCTA [12].

In the light of the above, this study investigated the detection of ICGA-confirmed polypoidal lesions by combining B-scan SS-OCTA and en face SS-OCTA-based characteristics in a Caucasian patient cohort.

## 2. Materials and Methods

### 2.1. Study Population

This retrospective observational case series included 32 eyes of 32 patients diagnosed with polypoidal lesions of different origins, independent of any prior treatment at our tertiary medical retina center (Medical Retina Unit, Department of Ophthalmology; Clinic Landstra*ß*e, Vienna Healthcare Group, Karl Landsteiner Institute for Retinal Research and Imaging, Austria) between June 2017 and March 2020. All patients underwent comprehensive ophthalmic examinations including dilated fundoscopy and multimodal imaging with SD-OCT, fluorescein angiography and ICGA (SPECTRALIS HRA-OCT Confocal Scanning Laser Ophthalmoscope and Angiography; Heidelberg Engineering, Heidelberg, Germany), SS-OCT, and SS-OCTA (DRI OCT Triton Plus; Topcon Corporation, Tokyo, Japan) as baseline standards of care on the day of admission. The best corrected visual acuity (BCVA) was measured using the Early Treatment Diabetic Retinopathy Study (ETDRS) letter score (4 m) and converted to Snellen (Sn). The SS-OCTA device works at a wavelength of 1050 nanometers under an acquisition rate of 100.000 A-scans per second with a motion contrast algorithm called OCTARA™ [13]. It operates on 1 mW input power with a digital axial resolution of 2.6 *µ*m and a transverse digital resolution range of 9.4 to 18.8 *µ*m, depending on the selected cube. Two trained operators captured SS-OCTA B-scans and standardized 4.5 × 4.5 mm, 6 × 6 mm^2^, and 9 × 9 mm^2^ en face SS-OCTA macular cubes with a scan resolution of 320 × 320 or 512 × 512 B-scans for each eye. The study adhered to the tenets of the Declaration of Helsinki. All subjects provided informed consent to analyze their data retrospectively and, hence, to participate in the study at the first presentation. The Federal Hospitals Act *§*15a Abs. 3a states that approval from the Viennese ethics committee is not needed for this study design.

### 2.2. Imaging and Grading

**Figure 1:**
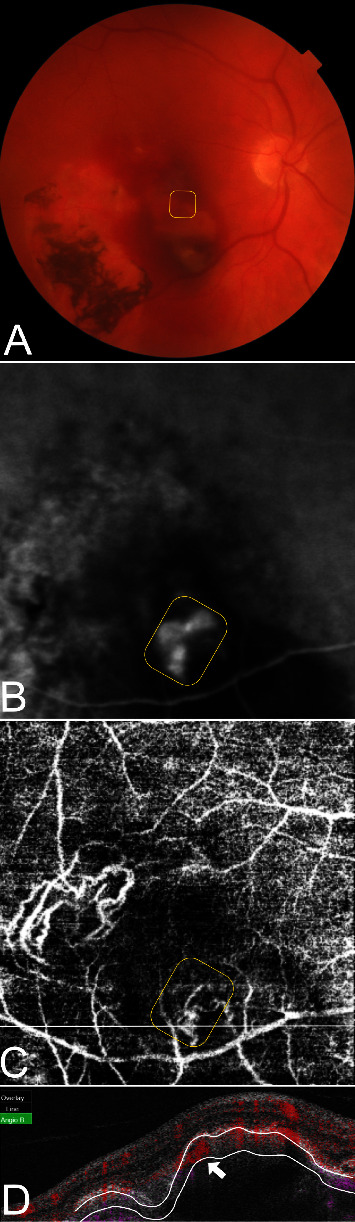
A solitary polypoidal lesion as a tangled vessel ball in en face SS-OCTA and as circumscribed flow in the B-scan SS-OCTA (a) retinal hemorrhage partially obscuring the polypoidal lesion (encircled) in color fundus imaging; (b) ICGA revealed a hypercyanescent spot (encircled) inferior to the fovea, corresponding to (c) a tangled vascular configuration (encircled) in 4.5 × 4.5 mm en face SS-OCTA; and (d) circumscribed flow (arrow) in B-scan SS-OCTA.

**Figure 2:**
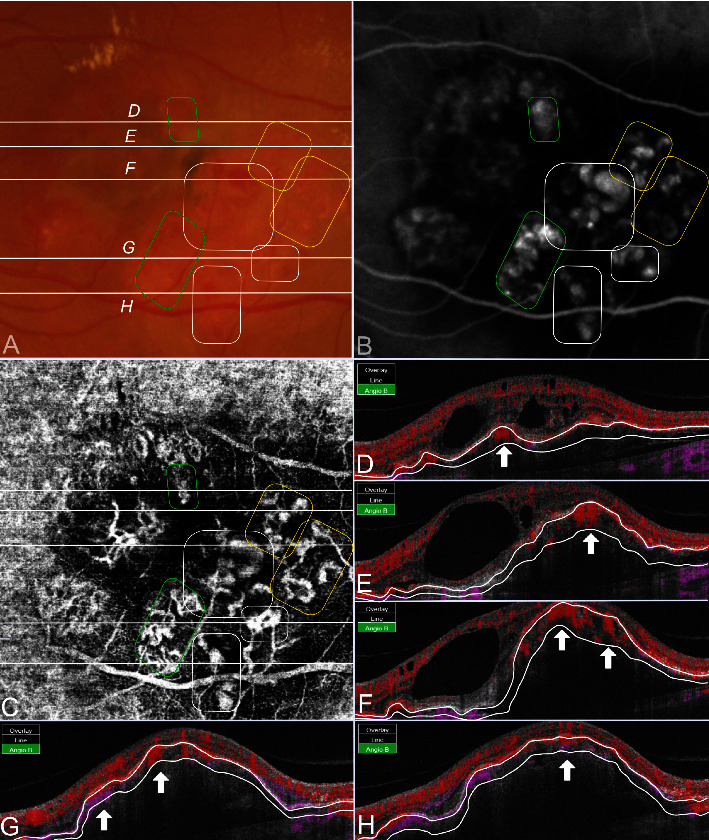
A cluster of polypoidal lesions as solitary tangled vessel balls, tangled vessel balls next to vascular dilatations, and dilatations only in en face SS-OCTA with circumscribed flow in B-scan SS-OCTA (a) color fundus imaging with red-orange nodules and exudation (b) ICGA identified 7 distinct clusters of polypoidal lesions besides the branching neovascular network, corresponding to (c) 2 tangled vessel balls (encircled in green), 2 tangled vessel balls with vascular dilatations (encircled in yellow), and 3 vascular dilatations only (encircled in white) in a 6 × 6 mm en face SS-OCTA scan. All lesions were identified as (d–h) circumscribed flow (arrows) in B-scan SS-OCTA.

**Figure 3:**
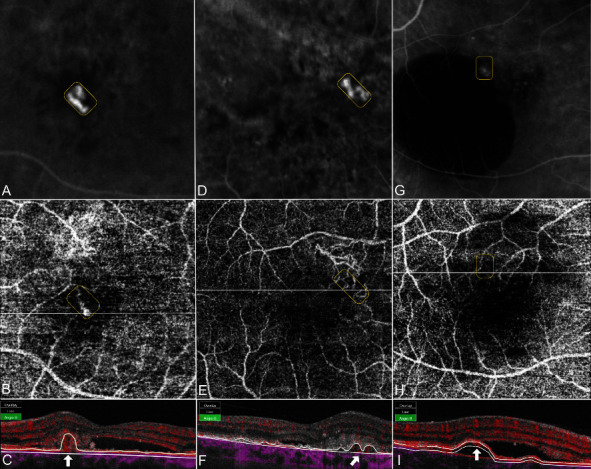
Polypoidal lesions either as a vascular dilatation only, as an ill-defined vascular network with diffuse flow, or as nondetection by en face SS-OCTA. (a) A polypoidal lesion (encircled) demarcated by ICGA as compared to (b) a vascular dilatation (encircled) in en face SS-OCTA with (c) diffuse flow (arrow), all-encompassing the PED in B-scan SS-OCTA. (d) A polypoidal lesion (encircled) clearly demarcated by ICGA (e) as compared to an ill-defined vascular network (encircled) using 4.5 × 4.5 mm en face SS-OCTA and (f) diffuse flow (arrow) within the PED in B-scan SS-OCTA. (g) An ICGA confirmed polypoidal lesion (encircled) with (h) detection failure (encircled) in 4.5 × 4.5 mm en face SS-OCTA but (i) circumscribed flow (arrow) within a PED in B-scan SS-OCTA.

Polypoidal lesions and the BNVN were diagnosed based on the EVEREST criteria and termed as suggested by medical retina experts [3, 14]. Two independent graders who were blinded to the angiographic findings evaluated all acquired SS-OCTA images for flow detection in B-scans and the structural morphology in en face scans by using the integrated OCTA analysis software IMAGEnet 6 (Version 1.24.1.15742, Topcon Corporation, Tokyo, Japan). The SS-OCTA grading approach was based on qualitative parameters for feasible reproducibility in clinical settings. In cases of grading disagreement, the images were independently reevaluated. If a grading disagreement existed, a senior clinical advisor was consulted. The final grade was determined by the majority vote of all three retinal specialists. The segmentation protocol defined the retinal pigment epithelium (RPE) as the inner line and Bruch's membrane (BM) as the outer segmentation boundary. In the case of segmentation artifacts, resegmentation was performed either semi-automatically by altering the predefined sections or by drawing the lines manually. First, SS-OCTA B-scans were investigated to identify flow inside various configurated PEDs [15]. Color-coded flow signals were either found to be circumscribed with a focal increase of density existing in various shapes and forms within the PED (Figures 1 and 2) or diffuse as scattered flow along the PED or as complete flow within the PED (Figure 3). Circumscribed flow was defined by the presence of increased flow signals within a focal area inside the PED, while every other abnormal flow without precise localization was determined to be diffuse.

The corresponding en face SS-OCTA images were superimposed with the hypercyanescent spots in ICGA by an alignment of the inner retinal vasculature to confirm the topography of the respective structures. After locating the vascular complex as the lesion of interest correctly, a number of different en face SS-OCTA patterns were elaborated: First, the previously described tangled vessel ball type (Figure 1), a neovascularization with convoluted vessels in a ball-like formation. Second, a tangled vessel ball adjacent to dilated vessels inconsistent with the BNVN (Figure 2) was detected. Third, single vascular dilatations were defined by the presence of larger vessel calibers exceeding the size of other pathologic vessel formations (Figures 3(a)–3(c)). Fourth, neovascular lesions without a tangled ball-shaped appearance or abnormal dilatations but unclear demarcation from the BNVN were termed as ill-defined vascular networks (Figures 3(d)–3(f)). More than 50% of the polypoidal lesion area had to be consistent with the respective morphological pattern for valid grading. On structural OCT B-scans, the PED height, configuration, and the localization of the polypoidal lesion inside the PED were analyzed as covariables. In addition, subfoveal choroidal thickness (SFCT) was measured as the greatest vertical distance between the BM and sclerochoroidal interface to differentiate the pachychoroid.

### 2.3. Statistics

The sensitivity of each investigated SS-OCTA imaging modality was calculated in comparison to ICGA. Due to the small numbers, the differentiation between scattered flow along the PED and complete flow within the PED was set in contrast to circumscribed flow on B-scan SS-OCTA. The relationship between B-scan SS-OCTA and en face SS-OCTA was evaluated using the chi-square test. Age, sex, laterality, underlying disease, BCVA, SFCT, PED height, PED configuration, lesion localization inside the PED, presence of hemorrhage, and previous treatment with intravitreal antivascular endothelial growth factor (anti-VEGF) were investigated as potential influencing factors using generalized mixed-effect regression analyses for the binary dependent variable B-scan SS-OCTA (comparing the probability for diffuse flow vs. circumscribed flow) as well as for the binary dependent variable en face SS-OCTA (comparing the probability for tangled vessel balls vs. other results). All models were accounted for with random factors for patients. Statistical significance was set at *p* < 0.05. Intergrader variability was calculated using Cohen's kappa coefficient (K) and corresponding 95% confidence intervals (CI). All analyses were performed using *R*, release 4.0.3, and SAS 9.4 software (SAS Institute Inc., Cary, NC, USA). The tables were illustrated using Microsoft Excel 2019 (Microsoft Corporation, Redmond, WA, USA). Figures were composed using Photoshop CC 14.0 (Adobe Systems Incorporated, San Jose, USA).

## 3. Results

In total, 43 polypoidal lesions were identified by the ICGA and were hence eligible for enrollment. The descriptive data and patient information summary are displayed in Tables 1 and 2.

**Figure 4:**
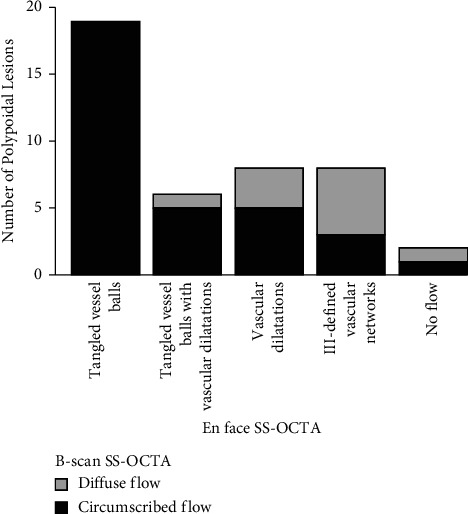
Distribution of B-scan swept source optical coherence tomography angiography (SS-OCTA) and en face SS-OCTA characteristics.

All polypoidal lesions in ICGA were also displayed by color-coded flow signals (100%). In detail, circumscribed flow within the PED was found in 33 (76.7%) polypoidal lesions (Figures 1(d), 2(d)–2(h) and 3(i)), while diffuse flow was observed in 10 (23.3%) lesions (Figures 3(c) and 3(f)). Vascular morphology was captured by en face SS-OCTA in 41 (95.3%) polypoidal lesions (Figures 1(c), 2(c), 3(b), and 3(e)), leaving 2 (4.6%) undetected (Figure 3(h)). Out of these, 19 (44.2%) were shaped as tangled vessel balls (Figure 1(c)), 6 (13.9%) as tangled vessel balls in addition to dilated vessels (Figure 2(c)), 8 (18.6%) as vascular dilatations only (Figure 3(b)) and 8 (18.6%) as ill-defined vascular networks (Figure 3(e)). A significant relationship between circumscribed flow in B-scan SS-OCTA and tangled vessel balls in en face SS-OCTA was evaluated (*p*=0.005) (Figure 4 and Table 3).

No multivariate analysis for potentially influencing cofactors was performed, as the univariate analysis showed no statistical significance (Table 3). Nonetheless, a statistical tendency of male sex (*p*=0.0783) and better BCVA (*p*=0.0778) were related to diffuse flow in B-scan SS-OCTA. In general, 37 of 43 (86%) flow patterns in B-scan SS-OCTA and 32 of 43 (80%) morphological patterns in en face SS-OCTA were graded equally. Good initial intergrader agreement was observed in both B-scan SS-OCTA (Κ = 0.65; CI = 0.40–0.91) and en face SS-OCTA (Κ = 0.68; CI = 0.52–0.85).

## 4. Discussion

In this study, the diagnostic value of SS-OCTA in polypoidal lesions regarding functional characteristics of flow signals and morphology was investigated and set in contrast to the invasive imaging modality ICGA. All lesions seen in ICGA were identified using color-coded B-scan SS-OCTA. Fujita and colleagues primarily focused on the analysis of B-scan SS-OCTA and presented a detection rate of 94.7% with typical flow characteristics in Asians [9]. They described polypoidal lesions in B-scans as complete or incomplete round/ring-like or flow signals adjacent to a PED notch. While the authors emphasized on a detailed description of the distributed flow, this study attached less importance to the morphology of flow patterns in B-scan SS-OCTA. Instead, flow density was described as an expression of functionality, either circumscribed by a focal area of high flow or diffuse without exact localization within the PED. Interestingly, a statistical tendency for male preponderance and better BCVA was related to a diffuse distribution, suggesting a heterogenic variability in the respective characteristics.

Circumscribed flow in B-scan SS-OCTA was significantly related to the appearance of tangled vessels balls in en face SS-OCTA (Figure 4 and Table 3). In fact, all tangled vessel balls observed in this study were visualized as circumscribed flow in B-scan SS-OCTA, which was attributed to the densely packed multiple vascular branches in tangled vessel ball formations. In total, en face SS-OCTA was inferior to color-coded B-scan SS-OCTA as it was not able to visualize the morphology of all ICGA-confirmed spots. Tangled vascular structures with or without dilatations besides ill-defined vascular structures were observed. These findings partly coincide with the observation of Bo and colleagues who first reported that polypoidal lesions in ICGA correspond to tangled vessel balls using en face SS-OCTA [10]. In their Asian study population, all confirmed polypoidal lesions were pictured as tangled vascular structures by en-face SS-OCTA. The authors concluded that polypoidal lesions are a distinct form of neovascularization rather than an aneurysmal structure. On the other hand, the BNVN was considered a variant of MNV type 1 and the term “aneurysmal” was found to be more appropriate than “polypoidal” to describe the vascular dilatations observed by multimodal imaging [16]. While retinal experts widely agree upon the vascular—some even refer to as arterial – origin rather than solid fleshy polyps, consensus has not been reached whether polypoidal lesions are simple aneurysms or more complex vascular structures [17]. A Japanese study group detected tangled vascular structures in 58 of 72 (80.5%) polypoidal lesions using en-face SS-OCTA as a secondary outcome measure [9]. Nevertheless, they reported difficulties in determining detailed structural findings in the remaining polypoidal lesions, which were attributed to image resolution limits or low quality. In our cohort of Caucasian patients, a variety of morphological appearances besides tangled vessels balls were expressed such as vascular dilatations in connection with tangled vessels, mere findings, and even ill-defined vascularity without a specific pattern. This complexity suggests a more heterogenic structure of polypoidal lesions and could very well be the manifestation of the same disease in a different stage—treated or treatment-naive p.e.—or a distinct subtype. One hypothesis is based on the transformation of neovascular patterns following repeated intravitreal antiVEGF injections. Therefore, prior antiVEGF treatment was analyzed as an influencing factor without significant findings. Other reasons including higher luminal pressure, focal vessel wall weakness, and genetic variability could be responsible for the differing appearances. The question remains as to whether dilated vessels are possibly aneurysmal neovascularizations as polypoidal lesions typically but not solely arise secondary to the pachychoroid disease spectrum.

The presence of hemorrhage had no impact on the appearance of polypoidal lesions. It is well known that polypoidal lesions pose a risk of extended subretinal hemorrhage as frequently seen in our study cohort (Figure 1(a)). ICGA, as a validated method, has a limited capability to detect polypoidal lesions underneath blood due to its attributes in the blood circulation. The noninvasive SS-OCTA technique highlights blood flow as void signals below the RPE in B-scans but also as vascular structures in en face scans. The herein detected relation between circumscribed high flow signals and tangled vessel balls could be useful to narrow the topographic localization of polypoidal lesions and might be preferential in the presence of subretinal hemorrhage, in the case of a strategical therapeutic change, for example. A prior anti-VEGF treatment was also investigated as a cofactor for the occurrence of morphologic patterns, but it showed neither a significant influence nor a tendency at the time of observation. The transformation of a polypoidal lesion into a MNV type 1 after previous antiVEGF treatment was not investigated in this study [18]. Structural OCT demonstrated a high diagnostic accuracy by applying a combination of 3 OCT-based criteria: sub-RPE ring-like lesions, en face OCT complex RPE elevation, and a sharp-peaked PED [3]. In our study, the identified polypoidal lesions were set in relation to the PED height and PED formation as pictured by OCT B-scans. All polypoidal lesions were elucidated below the RPE in B-scan OCTA. They were either located within a sharp-peaked PED (Figure 3(c)), below the top of a PED (Figures 2(e), 2(f), and 2(h)) or adjacent to a PED notch (Figures 1(d), 2(d), and 2(g)). However, the univariate analysis of coexisting OCT B-scan features showed no significance to either of the OCTA findings. Other OCT-based criteria such as the subfoveal choroidal thickness of the affected and the contralateral eye were also investigated without significant relation. Attributes such as age or origin were separately investigated as cofactors without statistically significant results (Tables 2 and 3). This does not automatically imply that the different characteristics are independent of the etiologic nature of the underlying disease. It simply states that the herein applied covariables had no influence on the appearance of polypoidal lesions in SS-OCTA.

The limitations of this study are attributed to its retrospective nature. The majority of polypoidal lesions were found to be solitary while only 6 eyes showed multiple lesions. Arguably, the cluster-like appearances in the same eye were likely to demonstrate similar SS-OCTA characteristics as described by Bo when in fact they showed a heterogenic picture as displayed in Figure 2 [10]. Nonetheless, no conclusion could be drawn as only two eyes were affected by this multiplicity of polypoidal lesions. The consensual descriptions of morphological appearances were partially extracted from previous studies, which could lead to a grader's bias. Another limitation is the description of qualitative flow parameters in B-scans, namely, focal or diffuse flow. This lack of diversity was partially driven by the low image resolution and the number of investigated eyes. However, good initial grader agreement underlines the predefined morphological expressions of flow signals and vascular patterns. To the best of our knowledge, this is the first study investigating the feasibility of SS-OCTA in Caucasian patients diagnosed with polypoidal lesions by combining flow signals in B-scan OCTA and vascular patterns in en face SS-OCTA.

In conclusion, this study elucidates the value of SS-OCTA and contributes to the noninvasive ICGA-independent diagnostic characteristics of polypoidal lesions. Our findings suggest a more diverse appearance of vascular patterns as a spectrum of the disease in Caucasians.

## Figures and Tables

**Table 1:** Descriptive data of patients enrolled in this study.

Mean age (SD), years	72 (9.5)
Sex, number (%)	
Male	15 (46.9%)
Female	17 (53.1%)
BCVA	
ETDRS letters, mean (range)	0.7 (0.08–1.18)
Snellen equivalent	20/50
Mean SFCT (range), *µ*m	294 (38–425)
Mean PED height (range), *µ*m	305 (58–1088)
Retinal hemorrhage	11 (34.4%)
St.p. Anti-VEGF	12 (37.5%)

SD, standard deviation; BCVA, best corrected visual acuity; ETDRS = early treatment diabetic retinopathy study; SFCT,  subfoveal choroidal thickness; PED, pigment epithelium detachment, St.p. = status post; VEGF, vascular endothelial growth factor.

**Table 2:** Patient information summary.

Patient no	Sex	Age	Laterality	Origin	No. of polypoidal lesions in ICGA	B-scan SS-OCTA		En face SS-OCTA			BNVN	Max. PED height (*µ*m) of polypoidal lesions	SFCT (*µ*m)	Retinal hemorrhage	No. of previous intravitreal antiVEGF injections
						Circumscribed flow	Diffuse flow	Tangled vessel balls	Vascular dilatations	Ill-definedvascular networks					
1	M	63	OS	PCDS	1	−	+	−	+	−	+	76	330	−	0
2	M	66	OS	PCDS	1	−	+	−	+	−	+	98	333	−	1
3	M	74	OD	PCDS	1	+	−	+	−	−	+	176	346	−	0
4	M	75	OD	AMD	2	+	−	+	+	−	+	593	292	+	0
						+	−	+	+	−		333			
5	M	90	OD	AMD	1	−	+	−	−	+	+	313	195	−	0
6	F	76	OD	AMD	1	+	−	−	−	+	+	142	84	+	0
7	M	76	OS	AMD	1	+	−	+	−	−	−	267	164	−	0
8	F	51	OS	PCDS	7	+	−	+	+	−	+	855	425	+	6
						+	−	−	+	−		1088			
						+	−	+	+	−		953			
						+	−	−	+	−		633			
						+	−	+	−	−		571			
						+	−	−	+	−		887			
						+	−	+	−	−		390			
9	F	74	OS	AMD	1	+	−	+	−	−	+	474	38	−	0
10	F	74	OD	Idiopathic	1	−	+	−	−	+	−	145	170	−	0
11	M	81	OS	AMD	1	−	+	+	+	−	+	264	185	+	0
12	F	59	OD	PCDS	1	+	−	+	−	−	+	198	340	−	28
13	M	76	OD	AMD	1	+	−	+	−	−	−	158	154	−	13
14	M	76	OS	AMD	1	+	−	+	−	−	+	232	326	−	0
15	F	84	OS	AMD	1	−	+	−	−	+	+	411	169	+	0
16	F	52	OD	PCDS	1	+	−	−	−	−	+	165	394	−	8
17	F	70	OS	Idiopathic	2	+	−	+	−	−	−	169	240	+	0
						+	−	+	−	−		148			
18	M	71	OD	AMD	1	+	−	+	−	−	+	223	287	+	1
19	F	73	OS	PCDS	1	−	+	−	−	+	+	79	378	−	0
20	M	83	OD	AMD	1	−	+	−	−	−	−	58	68	+	19
21	F	83	OD	AMD	1	+	−	−	+	−	−	85	82	−	0
22	F	85	OS	AMD	2	+	−	+	+	−	+	214	332	−	26
						+	−	+	−	−		178			
23	F	64	OS	Idiopathic	2	+	−	+	−	−	+	240	259	−	0
						+	−	+	−	−	+	172	259	−	0
24	M	58	OS	PCDS	1	−	+	−	+	−	+	190	209	+	0
25	F	84	OS	AMD	1	+	−	+	−	−	+	97	192	+	0
26	F	79	OS	AMD	1	+	−	−	−	+	+	271	84	+	0
27	M	60	OS	PCDS	1	+	−	−	+	−	+	572	371	−	10
28	F	71	OS	PCDS	2	+	−	+	−	−	+	282	374	−	21
						+	−	+	−	−		108			
29	F	69	OD	PCDS	1	+	−	+	−	−	+	229	257	−	1
30	M	73	OS	PCDS	1	−	+	−	−	+	+	85	289	−	14
31	M	67	OS	PCDS	1	+	−	+	−	−	+	170	271	−	10
32	F	71	OD	AMD	1	+	−	−	−	+	+	143	165	−	0

SS-OCTA = swept source optical coherence tomography angiography; ICGA = indocyanine green angiography; BNVN = branching neovascular network; PED = pigment epithelial detachment; SFCT = subfoveal choroidal thickness; anti-VEGF = antivascular endothelial growth factor; M = male; OS = oculus sinister; PCDS = pachychoroid disease spectrum; OD = oculus dexter; AMD = age-related macular degeneration; F = female; Idiopathic = origin not classified as PCDS, AMD, inflammatory, choroidal osteoma, or choroidal melanoma.

**Table 3:** Univariate *p* values (and CI 95% for odds ratios) of covariables potentially influencing the characteristics of polypoidal lesions in SS-OCTA.

Variables	SS-OCTA B-scan (diffuse vs. circumscribed flow)	SS-OCTA en face (tangled vessel balls vs. others)
Sex	0.0783 (0.789–43.17)	0.8902 (0.191–4.295)
Laterality	0.6831 (0.084–5.372)	0.5011 (0.123–2.967)
Age	0.227 (0.959–1.174)	0.8347 (0.934–1.086)
BCVA at baseline	0.0778 (0.649–>999)	0.1587 (0.011–2.302)
Pachychoroid disease vs. AMD	0.999 (0.031–32.14)	0.5186 (0.114–3.196)
Idiopathic disease vs. AMD	0.777 (0.049–50.665)	0.5152 (0.130–46.514)
Presence of hemorrhage	0.9615 (0.128–7.137)	0.9848 (0.209–4.925)
Prior treatment with anti-VEGF	0.1591 (0.505–39.416)	0.4068 (0.108–2.649)
PED height	0.1509 (0.986–1.003)	0.6095 (0.997–1.004)
PED configuration 1 vs. 0^*∗*^	0.5869 (0.049–6.031)	0.4144 (0.317–13.056)
PED configuration 2 vs. 0^†^	0.6694 (0.026–11.69)	0.9601 (0.317–13.056)
SFCT	0.1653 (0.986–1.003)	0.1798 (0.997–1.012)
SFCT of contralateral eye	0.3875 (0.986–1.006)	0.1642 (0.997–1.014)

SS-OCTA = swept source optical coherence tomography angiography; BCVA = best corrected visual acuity; AMD = age-related macular degeneration; MNV = macular neovascularization type 1; anti-VEGF = antivascular endothelial growth factor; PED = pigment epithelial detachment; ^*∗*^PED configuration 0 = sharp-peaked PED; PED configuration 1 = below the top of a PED; ^†^PED configuration 2 = adjacent to a PED notch; SFCT = subfoveal choroidal thickness.

## Data Availability

DAB and SAS had full access to all the data in the study and took responsibility for the integrity of the data and the accuracy of the data analysis. The datasets used and/or analyzed to support the findings of this study are available from the corresponding author on reasonable request.
